# Molecular memories in the regulation of seasonal flowering: from competence to cessation

**DOI:** 10.1186/s13059-015-0770-6

**Published:** 2015-09-15

**Authors:** Fabian Bratzel, Franziska Turck

**Affiliations:** Max Planck Institute for Plant Breeding Research, Department of Plant Developmental Biology, Carl von Linne Weg 10, 50829 Cologne, Germany

## Abstract

Plants commit to flowering based on endogenous and exogenous information that they can remember across mitotic cell divisions. Here, we review how signal perception and epigenetic memory converge at key integrator genes, and we show how variation in their regulatory circuits supports the diversity of plant lifestyles.

## Introduction

Flowering at the appropriate season and age is crucial for reproductive success as open flowers are particularly sensitive to adverse climatic conditions and seed maturation is costly in terms of energy and nutrient consumption. Furthermore, cross-pollination depends not only on coordinated flowering between individuals of the same species but sometimes also on the presence of appropriate pollinators. Synchronization of the plant life cycle with the sequence of seasons depends on the detection of environmental signals such as photoperiod and temperature, of which in particular the latter requires integration over several weeks to provide robust information. In many plant species, the response to these external signals is gated by endogenous factors such as sugars and hormone levels that are directly or indirectly related to developmental age [1–3]. Thus, for plants to be able to make an informed decision to commit to flowering, the ability to acquire and remember information through longer time periods and across mitotic cell divisions is crucial.

In the following, we discuss the pathways dependent on photoperiod, aging and vernalization, which are commonly part of the decision-making process for flowering. We set these pathways in the context of land plant evolution as this sets the frame for the expected conservation of molecular mechanisms contributing to their regulation. We then summarize the state-of-the-art on the regulation of these pathways, mostly based on studies performed in the model plant *Arabidopsis thaliana*. Finally, we explain how plants have diversified the cross-talk and regulatory connections between these main flowering pathways to support their great diversity of lifestyles.

## Plant lifestyles and the decision to flower

**Fig. 1:**
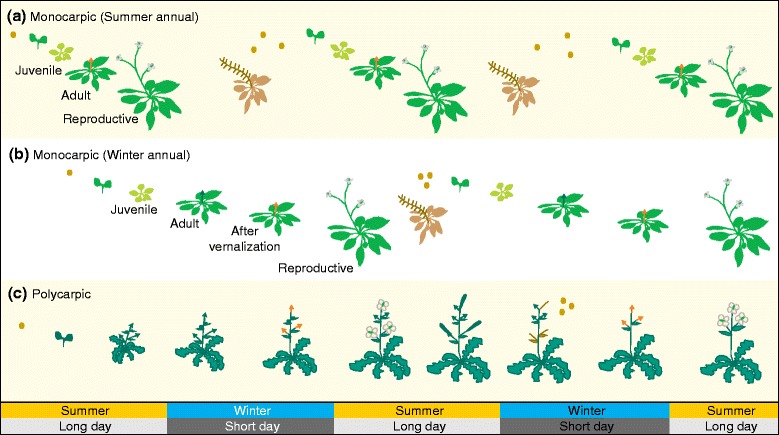
Schematic comparison of plant lifestyles. **a** Summer annual monocarpic plant. After germination in spring, flowering is induced within the same year as the plant is ab initio competent (*orange arrow*) to respond to flower-promoting long days. Each generation is terminated by seed development and subsequent senescence. **b** Winter annual monocarpic plant. After germination in spring, the plant only becomes competent to respond to floral induction after experiencing a prolonged period of cold (*green arrow*, vegetative shoot before vernalization; *orange arrow*, reproductive shoot after vernalization). **c** Polycarpic perennial plant. After germination in spring and after reaching maturity, a prolonged period of cold is required to respond to flower-inducing conditions. Main shoot meristems are vegetative before (*green arrows*), and become reproductive after vernalization (*orange arrows*). Reproductive transition is prevented for a subset of shoot meristems to ensure vegetative growth after flowering ceases until the following reproductive cycle. After seed production, reproductive shoots become senescent

All plant organs are formed from small populations of self-renewing stem cells, called meristems. When plants undergo the floral transition, shoot meristems *trans*-differentiate from a vegetative state, during which they produce leaves and lateral meristems, to a reproductive state, resulting in the production of inflorescences and flowers. To time flowering towards the appropriate season, photoperiod pathways track either day length or night length and, when a critical value is exceeded, induce florigen, a mobile signal produced in leaves that causes the reproductive transition in the meristems [4]. In *A. thaliana*, the transition from vegetative to reproductive development is controlled by interlinked molecular pathways that converge on the regulation of the flowering integrator genes *FLOWERING LOCUS T* (*FT*) and *SUPRESSOR OF CONSTANS 1* (*SOC1*). *FT* expression is induced in the leaf vasculature by long days (LDs), and the FT protein is the mobile florigen that moves to the shoot apex. In the apical meristem, FT and its binding partner, a bZIP transcription factor named FLOWERING LOCUS D (FD), trigger the transition from vegetative to reproductive growth by activating inflorescence/floral meristem identity genes such as *SOC1*, *FRUITFUL* (*FUL*) and *APETALA 1* (*AP1*) [2, 3]. To prevent floral transition before reaching maturity, the age-dependent pathway counteracts *FT* expression in the juvenile phase of vegetative development. In the case of *A. thaliana*, which is a facultative LD plant, prolonged age will eventually lead to flowering even in the absence of a promoting photoperiod. While many *A. thaliana* isolates (accessions) flower as summer annuals as described above, a large proportion adopts a divergent winter-annual lifestyle (Fig. 1). Winter-annual accessions require a prolonged period of cold before they can respond to flower-inducing LD conditions, a phenomenon referred to as vernalization. The vernalization requirement involves repression of *FT* by the MADS-domain transcription factor FLOWERING LOCUS C (FLC), which is gradually and irreversibly downregulated during vernalization by cold-induced chromatin modifications. As a typical monocarpic plant, *A. thaliana* flowers only once and completes its life cycle within one year by controlled senescence during seed set. By contrast, polycarpic perennial plants cease to produce flowers after a defined reproductive period and then resume vegetative growth until the next flowering season. This is usually achieved by preventing the reproductive transition of a subset of meristems, which then support vegetative growth until the following reproductive cycle. It is important to note that individual flowering shoots of many perennial plants are monocarpic, as they become senescent after flowering. Polycarpic plants tend to have a longer period of juvenility than monocarpic plants, during which they are incompetent to respond to flower-promoting signals (Fig. 1).

## The principles underlying molecular memories

To integrate endogenous and exogenous signals that occur sequentially, plant cells need to ‘remember’ acquired information across mitotic cell divisions. Importantly, these memories must be erased to re-establish sensitivity to external signals either in the next generation or within the same polycarpic individual during the next reproductive cycle (Fig. 1). Two types of molecular memories are relevant for flowering time regulation. The first is defined by the circuit structure of the flowering regulatory network, which contains many examples of ‘toggle switches’ and ‘feed-forward loops’ that ensure unidirectionality of the decision to flower [1, 5]. The second molecular memory is referred to as epigenetic and involves particular covalent modifications of chromosomal histone proteins and resulting local changes in chromatin structure [6]. However, not all chromatin modifications should be considered as epigenetic as they often reflect rather than define the expression state of their target genes [7]. Histone modifications associated with active gene expression are histone acetylation, histone H2B mono-ubiquitylation (H2Bub), histone H3 lysine 36 di-/tri-methylation (H3K36me2/3) and histone H3 lysine 4 tri-methylation (H3K4me3). These marks of the active state are deposited by a heterogenous group of enzymes collectively called Trithorax group (TrxG) proteins. Chromatin signatures associated with repressive states are set by Polycomb group (PcG) proteins and involve histone H3 lysine 27 tri-methylation (H3K27me3) and histone H2A mono-ubiquitylation (H2Aub). In *A. thaliana*, PcG proteins form two classes of Polycomb repressive complexes (PRCs) that catalyze histone modifications instrumental for gene repression and for inheritance of the repressive signature. PRC2 complexes are involved in the deposition of H3K27me3 [8]. PRC1 complexes are divided into at least two subclasses, of which canonical PRC1 complexes catalyze H2Aub, whereas it has been proposed that a non-canonical PRC1 compacts chromatin independently from H2Aub [8, 9]. Many examples in animals and plants show how the antagonistic action of TrxG and PcG complexes is involved in expression memory, but it is important to note that not all genes modified by either complex stably inherit their transcription state [6, 10].

## An evolutionary perspective on flowering pathways

**Fig. 2:**
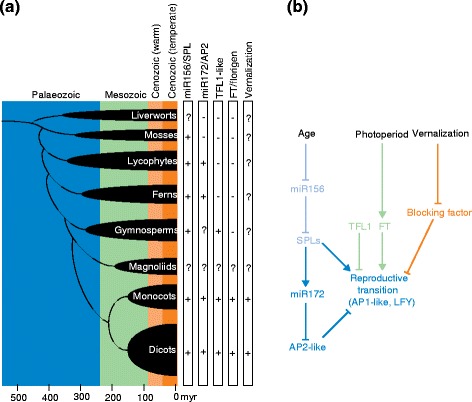
Evolution of flowering pathway networks in embryophytes. **a** Cladogram of embryophyte divisions highlighting the estimated evolutionary origin of flowering pathways during earth history. The embryophyte cladogram is plotted over the Phanerozoic (541–0 million years), and the recorded presence or absence of flowering pathway modules is indicated by *plus* or *minus signs*, respectively. A *question mark* indicates that no studies on flowering pathway modules are available yet. **b** Simplified flowering regulatory pathway network. The color code refers to the proposed evolutionary origin. The age-dependent pathway (*blue*) might be of Paleozoic origin as some of its components are conserved throughout all land plant divisions. The photoperiod pathway (*green*) probably evolved in the Mesozoic as homologs of florigen-encoding genes originated after angiosperms and gymnosperms separated. Vernalization pathways (*brown*) originated in the temperate Cenozoic as global cooling of the earth occurred only approximately 50 million years ago when land plant families had already separated. *Myr* million years

Flowering regulatory pathways can either accelerate or delay an intrinsic propensity of vegetative apical meristems to differentiate into inflorescences and flowers. In many examples, the age-dependent and vernalization pathways act to overcome an imposed delay or a repression of the transition, whereas photoperiod-dependent pathways tend to accelerate the process. How many pathways are active and how strongly they interact depends on the plant species and its adopted lifestyle. From an evolutionary perspective, the age-dependent pathway seems to be oldest as some of its components, such as *microRNA156* (*miR156*) and its corresponding target genes, are conserved throughout all land plants, including mosses [11] and liverworts [12] (Fig. 2). The pathway regulates not only reproductive but also vegetative transitions marking a shift from juvenile to adult traits — for example, increased complexity of leaf development and suppression of lateral branching in higher plants [13, 14]. By contrast, and in accord with the hypothesis that global cooling of the Earth occurred approximately 50 million years ago when land-plant families had already separated, the molecular nature of vernalization pathways differs between angiosperm families [15, 16] (Fig. 2).

The photoperiod pathway seems of intermediate age. *FT* homologs as florigen-encoding genes appear to have originated after angiosperms and gymnosperms separated. FT protein has been shown to act as a mobile florigen in monocot and dicot plant species [17–19]. Angiosperms contain a clade of *FT*-related genes that function as ‘anti-florigen’ and repress the reproductive transition in addition to affecting inflorescence architecture [20]. These genes are called *TERMINAL-FLOWER 1* (*TFL1*)-like, based on the corresponding *A. thaliana* mutant that forms a terminal flower almost immediately after germination in all photoperiod conditions [21]. Gymnosperms only feature genes equally related to *FT* and *TFL1* [22]. As the *FT* and *TFL1* genes of the spruce *Picea abies* repress flowering if expressed in *A. thaliana*, it has been argued that the repressive function is the more ancient one [22].

Despite their origin at the ‘root’ of angiosperms, the genetic modules implementing the functional connection between the clock/photoperiod and *FT* genes differ between plant families, although some gene families are more likely components [23]. In cases where homologous genes are involved in photoperiod control, as, for example, for the rice *CO* and *FT* orthologs *Heading Date 1* (*Hd1*) and *Hd3* [24, 25], the current state-of-the-art is to consider this as an example of convergent evolution.

## The regulation of florigen expression

**Fig. 3:**
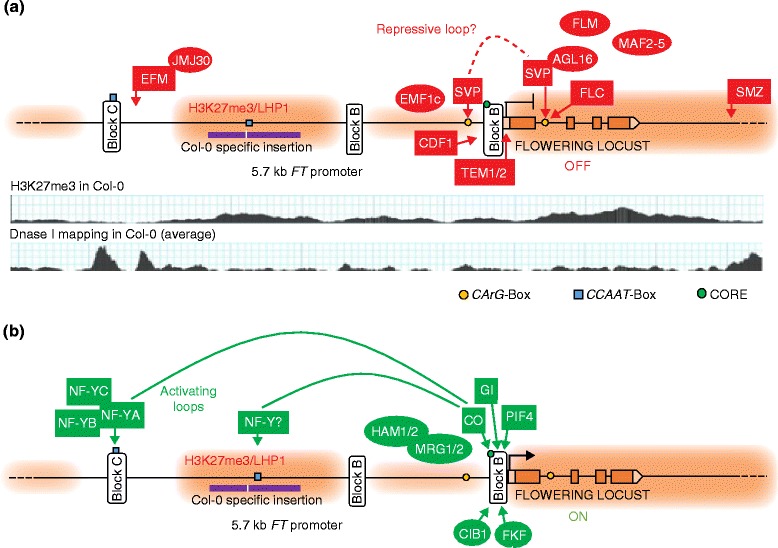
Regulatory modes of *FLOWERING LOCUS T* (*FT*). A 5.7-kb region upstream of the TSS is required for LD-mediated activation of *FT*. **a** Repressed state before floral induction. Repressive factors with evidence of DNA binding properties are depicted as *squares*, and their cognate DNA motifs are indicated. Repressive factors proposed to bind to chromatin marks or recruited by other factors are depicted as *ovals*. Below is diagrammed the H3K27me3 pattern [31] and DNase-I hypersensitivity profile [54] of the *FT* locus. Not mentioned in the text is CYCLIC DOF FACTOR 1 (CDF1), a protein that has been reported to repress *FT* expression through direct binding of the proximal promoter [2]. **b** Active state upon floral induction by LDs. Activating factors with evidence of DNA binding properties are depicted as *squares*. Activating factors proposed to bind to chromatin marks or recruited by other factors are depicted as *ovals*. Not mentioned in the text are GIGANTEA (GI), FLAVIN KELCH F BOX 1 (FKF1) and Cryptochrome Interacting Basic-helix-loop-helix 1 (CIB1), *FT*-inducing proteins that have been reported to bind directly to the proximal promoter [2]. *LD* long day. See main text for details of the protein identities

In *A. thaliana*, the photoperiod and vernalization signals integrate at the level of *FT* transcriptional regulation. *FT* expression is further modulated by ambient temperature and developmental age (Fig. 3). Although the molecular details of *FT* regulation might be less conserved than previously thought, the underlying principles of how transcription factor action is embedded in a chromatin landscape are likely to be common. The regulatory regions of *FT* do not only support the integration of a complex mixture of signals but they also define the hierarchy among promotive and repressive factors. Transcriptional activation of *FT* in LDs is predominantly controlled by the CCT domain transcription factor CONSTANS (CO), which, as for *FT*, is expressed exclusively in phloem companion cells [2, 3]. *CO* expression shows circadian oscillation, with a peak towards the end of the day. As CO protein is unstable in the dark, sufficient amounts to activate *FT* can only accumulate in LDs, when the presence of mRNA coincides with protein-stabilizing light.

### The role of three-dimensional chromatin structure in coordinating transcription factor action at *FT*

We will now describe the emerging concept regarding how different three-dimensional conformations of the chromatin might underlie signal integration at the *FT* locus. *FT* possesses a promoter that is unusually long for *A. thaliana*, and the gene also features regulatory elements in its introns. Upregulation of *FT* by CO is dependent on two regulatory regions that are conserved among *FT* orthologs within the Brassicaceae [26, 27] (Fig. 3). The proximal *FT* promoter contains several CONSTANS responsive elements [COREs; TGTG(N)_2–3_AT] that can be bound by CO in vitro [28] and impact *FT* expression in vivo [26, 29]. A second regulatory region, called BlockC, represents a distal enhancer located 5.7 kb upstream of the transcription start site (TSS). While the presence of both regions is necessary and sufficient for photoperiod control of *FT*, regions outside of these core control regions further modulate expression [27].

BlockC contains a CCAAT box that is crucial for *FT* activation in LDs [29]. CCAAT boxes are recognized by the NF-YA component of trimeric NF-Y transcription factors [30]. NF-YB and NF-YC components physically interact with CO [31, 32], and the NF-YA component and the CCT domain of CO show structural homology [31]. NF-Y components are encoded by small gene families, and the analysis of stacked mutants confirms that their presence is required for *FT* activation by CO [32, 33]. Two recent publications reported a chromatin loop between the proximal promoter and BlockC that was detected by application of chromatin conformation capture (3C). Chromatin looping explains communication between the distal regulatory element containing the canonical CCAAT box and the proximal promoter with its COREs [27, 29]. Decreased interaction frequency between BlockC and the proximal promoter correlated with reduced *FT* repression. This was observed in *co* and *nf-yb2; nf-yb3* mutants or in T-DNA lines with increased distance between the proximal promoter and BlockC [27, 29]. In addition, both studies detected strong interaction of the proximal promoter with intermediate regions, but the points of interaction differed between the studies. Further experiments are required to determine whether the 3C method, which was developed for long-range interactions of at least 10 kb, is adequate to interrogate chromosomal interactions reliably at shorter range. Nevertheless, it is an attractive hypothesis that chromosomal interactions between the distal enhancer and the proximal promoter create a poised environment that facilitates recruitment and stabilization of CO at proximal CORE elements (Fig. 3).

A complex formed by the MADS domain proteins FLC and SHORT VEGETATIVE PHASE (SVP) represses *FT* transcription [34]. Differential expression of *FLC* determines the response to vernalization (see below), whereas SVP acts early in development, in particular at low ambient temperature. At high *FLC* levels corresponding to the winter-annual lifestyle, the photoperiod response of *FT* is fully suppressed, whereas lower levels modulate the response. A small family of *FLC* paralogs, named *FLM* (*MAF1*) and *MAF2* to *MAF5*, participates in *FT* repression [35]. FLM, as for SVP, represses *FT*, particularly at low ambient temperature owing to a temperature-dependent shift in the equilibrium of *FLM* splice variants [36–38]. In addition, AGL16 associates with the SVP–FLC complex through a direct interaction with SVP, and, in the absence of AGL16, high levels of FLC cannot fully block *FT* induction in LDs [39]. Although not yet experimentally confirmed, MADS domain proteins are probably involved in three-dimensional chromatin interactions. FLC and SVP preferably bind to two regions containing CArG boxes in the first *FT* intron and intermediate promoter, respectively [34, 40]. As the region between the binding sites includes the *FT* proximal promoter, an AGL16–SVP–FLC-like interaction might antagonize interaction of the promoter with the distal enhancer BlockC.

The existence of three-dimensional interactions could also explain the mode of action of a second group of direct *FT* transcriptional repressors, which are related to APETALA2 (AP2) and commonly regulated through the age-dependent pathway (see below). Genetic data suggested that the clade member SCHLAFMÜTZE (SMZ) negatively regulates *FT* expression in leaves [41]. Genome-wide profiling of SMZ binding sites showed direct binding to many regulators of the floral transition, including *FT*; however, at *FT*, the binding site was located in a region 1.5 kb downstream of the gene [41]. Apart from a further possible three-dimensional interaction causing repression, it was speculated that SMZ impacted *FT* through the upregulation of *TEMPRANILLO 1* (*TEM1*) and *TEM2*, paralogs belonging to the RAV-clade of AP2-related factors [42]. *TEM2* expression was increased in response to increased SMZ levels [41], and direct binding of *TEM1* to the 5′-untranslated region (5′-UTR) of *FT* has been demonstrated [43]. Finally, repression of *FT* by *SMZ* is fully dependent on the presence of a functional copy of the MADS domain factor FLM, but there is no evidence of a physical interaction between the two factors [41].

### The role of chromatin modifications in regulating *FT*

Accurate timing of flowering relies on proper temporal and spatial control of *FT* expression in phloem companion cells of rosette leaves. Although the regulation of tissue specificity of *FT* expression is not yet fully understood, the role of PcG-mediated repression in the temporal control of expression has been described in detail [26, 44, 45]. The *A. thaliana* PcG mutants *embryonic flower 2* (*emf2*) and *emf1*, *curly leaf* (*clf*), *multicopy suppressor of ira 1* (*msi1*) and *like-heterochromatin protein 1* (*lhp1*) show an early to extremely early flowering phenotype in both LDs and short days (SDs), which is largely due to de-repression of *FT* [8, 9, 46]. EMF2, CLF and MSI1 are core subunits of EMF2–PRC2 complexes that create the repressive chromatin mark H3K27me3 [47]. LHP1, which features a chromodomain with affinity to H3K27me3, is reported to interact with PRC2 complexes and is also part of canonical and non-canonical PRC1 complexes [8, 46]. In addition, overexpression of *RELATED TO EARLY FLOWERING 6* [*REF6* (*JMJ12*)], which encodes a jumonji domain protein with H3K27me2/me3 de-methylase activity, results in increased *FT* expression and early flowering [48]. Different from the marks on most target genes in *A. thaliana*, H3K27me3 at the *FT* locus is not restricted to the gene body but spreads into the promoter and downstream regions [49]. While BlockC is free of the H3K27me3 modification and shows an open chromatin structure according to genome-wide DNase-I hypersensitivity profiling [50], the proximal promoter is rather inaccessible and positive for H3K27me3 (Fig. 3). This indicates that PcG-mediated repression acts mostly at the proximal promoter but less on the regulatory components present at BlockC. Indeed, deletion of the BlockC sequence silences *FT* expression in leaves of wild-type but not *lhp1* and *clf* mutant plants [26, 51].

It is expected that activation of *FT* involves at least temporal conversion of chromatin from a repressive to a permissive signature. Dynamic changes in H3K27me3 and the presence of chromatin modifications positively correlated with expression are difficult to detect at *FT* in wild-type plants probably owing to its restricted expression domain [26, 45, 52, 53]. Circumstantial genetic evidence shows how CO-mediated upregulation of *FT* expression involves chromatin factors. First, Morf Related Gene (MRG) chromodomain proteins MRG1 and MRG2 directly bind to the intermediate-proximal *FT* promoter [54, 55] in a manner dependent upon CO and histone H3K36me/H3K4me [54]. In situ proximity ligation assays suggest that MRG1 and MRG2 bind to H3K4me3 and H3K36me3 at many sites and, furthermore, associate globally with the H4-specific histone acetylases HISTONE ACETYLTRANSFERASE OF THE MYST FAMILY 1/2 (HAM1/HAM2) [56] to link H3K4me and H3K36me with histone acetylation [55]. At the *FT* locus, the interaction establishes an active chromatin signature of upregulation of *FT* expression by CO in LD conditions (Fig. 3).

As *FT* levels cycle in a diurnal pattern in LD conditions, chromatin modifications causing transcriptional upregulation need to be removed in the night. Removal of the active signature H3K4me3 by demethylation is mediated by the demethylase JMJ14 (PKDM7B) containing a jumonji C (JmjC) domain [52]. A recent study showed that JMJ14 is part of a PcG complex required for repression of *FT* in phloem companion cells during the night [57]. This non-canonical PRC1 complex, termed EMF1c, consists of the PcG members EMF1, LHP1 and JMJ14. The EMF1c complex is active in the leaf vasculature and represses *FT* downstream of the photoperiod pathway [57]. Taking into account these recent studies, a model of photoperiod-mediated *FT* induction emerges in which conversion of the *FT* locus from an FLC/FLM–EMF1–JMJ14-dependent repressive state to an active state is mediated through replacement of EMF1c by a CO–MRG1/MRG2–HAM1/HAM2 module at the promoter region.

Chromatin modifications are directly and indirectly involved in repression of FT mediated through the MADS domain factor. First, FLC and FLM interact with EMF1c, which might affect targeting of either complex to *FT* [57]. Second, SVP increases expression of the Myb domain factor EARLY FLOWERING MYB PROTEIN (EFM), which in turn plays a repressive function at the *FT* locus. EFM interacts with the H3K36 demethylase JUMONJI 30 (JMJ30) and, by binding to a region of the *FT* promoter downstream of BlockC, recruits JMJ30 to the *FT* locus, where it accumulates in the 5′ regions of the gene [58]. In *jmj30* or *efm* mutants, the H3K36me2 modification accumulates in the region associated with JMJ30, and *FT* expression is increased at its peak time in LDs.

### Expression of *FT* in a non-inductive photoperiod

Although the main signal for *FT* induction is LDs, *FT* expresses at sufficient levels to accelerate flowering if plants are grown at high ambient temperature in SDs [38]. Such a response could be adaptive as it might help plants to develop faster in an environment affected by drought. The *FT* response to high ambient temperature can in part be explained by the decreased repressive effect of SVP and FLM [36–38]. In addition, chromatin-mediated repression might be affected by ambient temperature more generally. Accordingly, an increase in ambient temperature leads to an eviction of nucleosomes containing repressive histone H2A.Z to allow binding of the activating basic helix-loop-helix (bHLH) transcription factors PHYTOCHROME INTERACTING FACTOR 4 (PIF4) and PHYTOCHROME INTERACTING FACTOR 5 (PIF5) to the proximal promoter region of *FT* [59, 60]. Genome-wide profiling showed that H3K27me3 and H2A.Z are highly correlated across the gene body of PcG target genes, but it is unclear whether this generally impacts their regulation at higher ambient temperatures [61].

Expression of *FT* in SDs is also observed in the phloem of developing siliques (fruits) [51]. Expression in this tissue is required to prevent reversion of the inflorescence meristem to a more vegetative state [51, 62]. Similar to the situation in PcG mutants, *FT* expression in siliques does not require the distal *FT* enhancer BlockC, indicating that a different enhancer region might be controlling *FT* expression at this late developmental stage [51].

### Regulation and roles of *FT*-like genes

In the course of evolution, plant families show a tendency to increase the number of genes encoding *FT* paralogs (for extensive reviews, see [4, 20]). Not only is this paralleled by an increased number of processes regulated by a photoperiod-controlled mobile signal, but it also leads to a more complex regulation of florigen expression. In *A. thaliana*, the only *FT*-paralog, *TWIN SISTER OF FT* (*TSF*), lacks the distal enhancer BlockC but shows extensive conservation of the proximal promoter [26]. *TSF* contributes only marginally to flowering time under controlled greenhouse growth conditions but colocalizes with quantitative trait loci (QTLs) for flowering time under field conditions [63, 64]. *TSF* is generally co-regulated with *FT* but expressed at much lower levels, which might be explained by either the absence of the enhancer or the presence of several heterochromatic transposable elements (TEs) in close proximity downstream [65]. TE insertions have also been linked to the loss of expression of one *FT* ortholog in *Brassica rapa*, which features six paralogs [66].

An interesting case of *FT* paralog neo-functionalization has been reported in sugar beet, where a limited number of point mutations converted one *Beta vulgaris FT* paralog from florigen to anti-florigen [67]. The negative regulator of flowering Bv*FT1* is expressed in SDs and in biennial accessions also in LDs before vernalization. The florigen Bv*FT2* is expressed only in LDs after Bv*FT1* expression has been permanently suppressed by vernalization.

## Acquiring the competence to respond to flower-promoting signals

Floral induction by photoperiod and/or high ambient temperature can be counteracted by the age-dependent pathway, which prevents flowering in juvenile plants that have not yet acquired enough resources and by the vernalization pathway that prevents flowering in late summer or autumn before vernalization. In the following section, we outline the molecular mechanisms underlying the vernalization and the age-dependent pathways in *A. thaliana*.

### Noncoding RNAs prevent precocious flowering

**Fig. 4:**
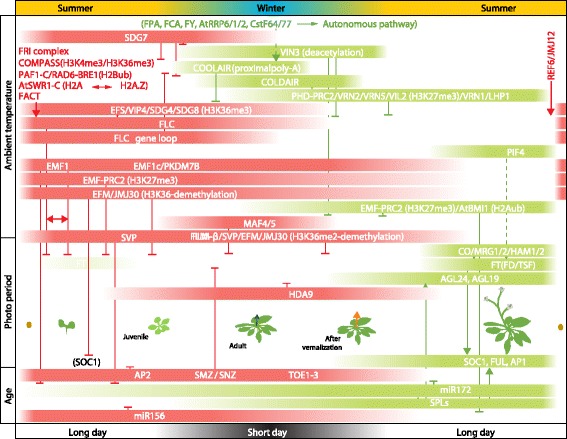
The age-, photoperiod- and vernalization-dependent flowering regulatory pathways of the winter annual monocarpic plant *Arabidopsis thaliana*. The seasonal activity of chromatin-related factors promoting flowering is depicted in *green*, and factors repressing flowering are shown in *red. Arrows* and *T-bars* indicate promoting and repressive effects on the target activity, respectively. If characterized, catalytic activities on the targets are indicated in *brackets*. See main text for a detailed explanation of the pathways. *Green arrow*, vegetative shoot before vernalization; *orange arrow*, reproductive shoot after vernalization

Two connected microRNA (miRNA) and target transcription factor modules act as developmental timers in many plant families (Figs. 2 and 4) [68]. Several excellent recent reviews have discussed the topic in detail [68–70], which allows us here to focus on points most relevant in the context of molecular memory. The first module in the age-dependent pathway comprises eight genes encoding *miR156*, which target mRNAs of several SQUAMOSA-BINDING-PROTEIN-LIKE (SPL) transcription factors for degradation and/or suppress their translation [13, 71, 72]. In *A. thaliana*, SPL transcription factors can promote flowering directly by inducing the expression of positive regulators of the reproductive transition, such as the MADS domain transcription factors *SOC1*, *FUL* and *AP1*, which are most effective in meristems [72, 73]. SPLs also positively regulate *FT* in the phloem companion cells of the leaves [72, 74]. As the FT–FD complex also positively regulates the expression of *SOC1*, *FUL* and *AP1*, *miR156* is the basis for a feed-forward loop inducing the reproductive transition [75]. Furthermore, *FT*, *SOC1*, *FUL* and *AP1* are also indirectly regulated by SPLs, which induce the expression of genes encoding *miR172* [75]. *miR172* RNAs target AP2 and the related factors SMZ, SCHNARCHZAPFEN (SNZ) and TARGET OF EAT 1 to TARGET OF EAT 3 (TOE1–TOE3), which collectively act as direct transcriptional repressors of genes promoting the floral transition [41, 76, 77]. In many plant species, expression of *miR156* genes is gradually downregulated during the first weeks of development [14, 78–80]. In *A. thaliana*, the MADS domain transcription factors AGL15 and AGL18 form a heterodimer that might directly activate transcription of primary (*pri*)-*mirRNA156a*/c. Accordingly, *agl15;agl18* double mutants show an early-flowering phenotype [81]. As AGL15 expression is directly activated by AP2, a regulatory feedback system connects the start and end-point of the regulatory cascade [77].

Expression of *miR156* and *miR172* as well as their target loci is likely to be influenced by the PcG pathway, given the high number of H3K27me3-positive loci within the network [82]. A recent study demonstrates that the H2Aub and H3K27me3 levels at the TSS of *miR156a* and *miR156c* are dependent on the function of AtBMI1A and AtBMI1B, which are part of canonical PRC1 complexes. Age-dependent silencing of *pre-miR156a* and *pre-miR156b* is impaired in *atbmi1a atbmi1b* double mutants, leading to a prolongation of the juvenile phase [83]. By contrast, the PcG members EMF1 and EMF2 are required for maintenance of *miR172* repression in the juvenile phase [83].

### Vernalization removes a block to flowering

To prevent flowering in unfavorable seasons, such as cold winters or dry summer periods, many plant species possess an effective block for their response to promotive signals that has to be removed by vernalization [15]. In the Brassicaceae family, the MADS domain transcription factor FLC and its orthologs implement this block by directly repressing genes that are positive regulators of the floral transition, such as *SOC1* in the shoot apex and *FT* in the phloem companion cells of leaves [40] (Fig. 4). High levels of *FLC* expression are dependent on the activity of the FRIGIDA complex (FRI-C) that is recruited by EARLY FLOWERING IN SHORT DAYS (EFS) [84] and consists of the FRI-like factors FRL1 and FRL2, FRIGIDA ESSSENTIAL 1 (FES1), SUPPRESSOR OF FRIGIDA 4 (SUF4), FLX and FLL4 [85, 86]. FRI-C forms a scaffold for recruitment of generic transcription factors that are involved in establishing an active chromatin signature at *FLC* [85, 87]. Induction of *FLC* transcription requires recruitment of the pre-initiation complex (PIC) comprising RNA polymerase II (RNAP II) as well as general transcription factors and accessory factors [88]. Assembly of the PIC is accompanied by a replication-independent substitution of histone H2A by H2A.Z-containing nucleosomes around the TSS, a process implemented by the ATP-dependent chromatin-remodeling complex AtSWR1-C. It has been proposed that AtSWR1-C is recruited by FRI-C as some subunits directly interact with three AtSWR1-C subunits [85, 89]. Transcriptional initiation also involves recruitment of RNA polymerase II associated 1 complex (AtPAF1-C), which is involved in recruitment of histone chaperones, as well as ubiquitylation complex subunits RAD6–BRE1 that catalyze H2Bub [53, 90]. H2Bub in turn is required for recruitment of proteins with homology to yeast complex proteins associated with Set1 (COMPASS) subunits [90]. The AtCOMPASS-like complex contains TrxG-related SET1 domain proteins implicated in catalysis of the H3K4 methylation that, together with H2Bub, accumulates around the TSS [91]. An active chromatin signature of *FLC* also requires the catalysis of the co-transcriptional H3K36me2/3 mark at the gene body, implemented by SET2 domain histone methyltransferases [92].

After a shift to chilling temperatures, a drop in *FLC* mRNA transcriptional activity is accompanied by transcription of noncoding RNAs (ncRNAs) from the *FLC* locus (Fig. 4). First, several antisense ncRNAs, collectively termed *COOLAIR*, are transcribed from a promoter located downstream of the *FLC* 3′ UTR [93]. As transcription of *COOLAIR* and *FLC* decreases with time, a sense ncRNA termed *COLDAIR* is induced from the first intron of the *FLC* locus [94]. Finally, *FLC*, *COOLAIR* and *COLDAIR* transcripts become fully silent upon prolongation of cold. Transcriptional shutdown mediated by antisense transcription has been linked to long-term epigenetic silencing that involves the replacement of the active chromatin mark H3K36me with the repressive mark H3K27me3 at the *FLC* locus [95, 96].

In parallel to an active reduction of H3K36me3, the activity of PRC2 complexes organizing the repressive H3K27me3 chromatin signature at *FLC* is increased during vernalization [96]. Epigenetic repression of *FLC* is dependent on the PRC2 component VRN2, which presumably replaces EMF2 to form a VRN2–PRC2 complex [87]. Moreover, the pleckstrin-homology domain (PHD) protein VIN3 associates with PRC2 complexes, which is thought to stimulate the activity of PHD–PRC2 complexes [97, 98]. *VIN3* expression peaks towards the end of exposure to cold and rapidly drops as the temperature rises. *VIN3* paralogs *VEL1* and *VRN5* are constitutively expressed and participate in *FLC* silencing by replacing VIN3 at *FLC*.

Among other possible mechanisms, the VRN2–PRC2 complex is recruited to the *FLC* locus by *COLDAIR*, which has been shown to bind to the VRN2–PRC2 subunit CLF [94]. *COLDAIR* recruits VRN2–PRC2 to a region close to its TSS in intron 1 of *FLC*, whereas the nucleation site for increased H3K27me3 during chilling is located at the TSS of *FLC* [99, 100]. At this nucleation site, H3K27me3 levels increase quantitatively during the cold period, whereas subsequent spreading of H3K27me3 throughout the entire *FLC* locus occurs as the ambient temperature increases [101]. How H3K27me3 spreading is mediated is not entirely clear, but the process depends on the presence of the constitutively expressed B3 domain protein VRN1 as well as the presence of the PcG component LHP1 [102–104].

A number of seminal studies demonstrated that stable maintenance of repression of *FLC* is a quantitative process involving a cell-autonomous bi-stable chromatin switch [99, 101, 105, 106]. Accordingly, in each relevant cell, the active H3K36me3 mark at the locus is replaced by the antagonistic active mark H3K27me3, whereby the probability to switch increases with the duration of cold exposure [96]. Long exposure to cold thus results in an increasing number of cells that have switched off *FLC* transcription, ultimately resulting in a release and thus increasing expression of the systemic flower-promoting signal *FT*. It was shown that not only is the memory of cold itself ‘digitally’ registered, but already the registration of cold exposure, namely the initial accumulation of H3K27me3 at *FLC* locus nucleation sites, is mediated in an all-or-nothing fashion in order to allow a robust and strict response to given natural fluctuations of temperature [101].

A yet-unanswered question is how perception of low temperature is mechanistically transformed to trigger the process of *FLC* downregulation during vernalization. It was recently discussed how reorganization of the chromatin topology in response to cold might provide a mechanism of thermodynamic control [107, 108]. Indeed, a gene loop between *FLC* 5′ and 3′ flanking regions is disrupted by cold. This process was paralleled by *FLC* downregulation and *COOLAIR* antisense transcript upregulation [109, 110]. Thus, cold-induced thermodynamically mediated reorganization of chromatin topology might release the *COOLAIR* promoter and facilitate higher antisense transcription to initiate downregulation of *FLC*.

### Interlocking of juvenility and vernalization modules regulates seasonal flowering in perennial Brassicaceae

Expansion of research to other Brassicaceae has revealed common principles as well as different strategies of flowering time regulation in the *A. thaliana* relatives *Arabis alpina* and *Cardamine flexuosa*. The *A. alpina FLC* ortholog *PERPETUAL FLOWERING 1* (*PEP1*) is downregulated during vernalization, which allows ‘mature’ shoot meristems to transition to inflorescences that form floral primordia during the cold period [111, 112]. An inductive photoperiod is not required for the floral transition in this species. Juvenile plants containing only immature meristems do not transition when vernalized [85]. Although young meristems do not flower, they respectively downregulate and upregulate *PEP1* and *AaSOC1* in the cold. The inability of SOC1 to trigger the transition is in part explained by higher levels of the anti-florigen *AaTFL1* in young versus more mature meristems [85]. In addition, *PEP2*, a paralog of *miR172*-controlled *AP2*, is involved in the regulation. Loss of *PEP2* function causes a phenotype similar to that of *pep1* mutants, namely flowering without a requirement for vernalization and without seasonal cessation — thus, perpetually [79]. In wild-type *A. alpina* plants, *AP2* and *miR172* are controlled by both age and vernalization as prolonged cold increases *miR172* levels only in mature plants but not in juvenile plants that express still high levels of *miR156*. A decrease in *miR156* and a subsequent increase in a group of *SPL* genes are observed as meristems age. Thus, in *A. alpina*, the decrease of *miR156* is not directly coupled to an increase in *miR172* but further requires vernalization, whereas vernalization is ineffective before the decrease of *miR156*.

Regulation in the related perennial species *C. flexuosa* shows a variation in the connection between the aging and vernalization pathways. Here, the coupling between the *miR156–miR172* regulons seems direct and not further gated by vernalization [80]. Furthermore, in *C. flexuosa*, increased *SOC1* expression is correlated with inflorescence development, in contrast to *A. alpina*, where both can be uncoupled.

As indicated by their name, perpetual flowering *pep* mutants flower continuously once they have passed the juvenile stage. Thus, *PEP1* and *PEP2* play a dual role in preventing the floral transition before vernalization as well as beyond the end of the reproductive season. *PEP1*, in contrast to its annual ortholog *FLC*, is not stably silenced after vernalization but upregulated after the return of warmer temperatures [112]. Differences in the H3K27me3 chromatin state of both genes have been identified, but further studies are required to separate causes and consequences. *COOLAIR* antisense transcripts are induced in both species in the cold, whereas *COLDAIR* is not detected in *A. alpina* [113]. However, downregulation and recruitment of H3K27me3 occurs in both species, indicating that differences might be more related to the maintenance of the epigenetic state than to its setting. In fact, *PEP1* shows a much higher and broader increase of H3K27me3 during the cold than *FLC*, for which the increase is mainly restricted to the nucleation region at the TSS [108]. Differences are more striking after the return to warm temperatures, when H3K27me3 levels rapidly decrease at *PEP1*, whereas they increase further across the gene body at *FLC* [112].

It is not yet known whether differences in *PEP1* and *FLC* regulation are explained by *cis* or *trans* effects. A genomic *PEP1* fragment that was stably repressed after vernalization was poorly expressed in *A. thaliana* even before vernalization, suggesting that both *cis* and *trans* differences exist between the species. Candidates for *trans*-components are, for example, the more specialized PcG components *VRN2* and *VRN1* [114], as well as components of the FRI-C required for high *FLC* expression. In *A. thaliana*, resetting of the epigenetic memory of *FLC* occurs in the embryo and is dependent on the presence of TrxG components [115–117] and EARLY FLOWERING 6 (ELF6), a jumonji-domain demethylase related to REF6 [118].

## Vernalization and chromatin in temperate grasses

Flowering pathways in temperate grasses (Poaceae) diverge from the established Brassicaceae models. The requirements for photoperiod induction and vernalization are common and well investigated in the grasses barley, wheat and *Brachypodium distachyon* (purple false brome), making it worthwhile to discuss differences and commonalities. The impact of the aging pathway on flowering is less studied in temperate grasses, but there is some indication that it participates in the response to ambient temperature in *B. distachyon*. In this species, the expression of *miR156* is upregulated in transgenic lines possessing reduced levels of *BdVIN3-LIKE 4* [119]. The upregulation of *miR156* was stronger in plants grown at low ambient temperature and correlated to late flowering. *BdVIL4* encodes a PHD domain protein related to *A. thaliana* VIN3, which is required for the epigenetic memory of *FLC* repression during vernalization [97].

The three main players of the photoperiod-mediated flowering response in barley are the pseudo-response regulator CCT domain protein PPD1, a barley CO homolog and the FT-related protein VRN3. Barley *CO* regulation is mediated through the circadian clock, involving PPD1, and CO protein levels are highest and sufficient for *VRN3* induction at the end of LDs, which is similar to the situation for *A. thaliana* [120]. Winter barley varieties additionally require a long exposure to low temperatures to respond to flower-inducing LDs, and a trio of genes related to *AP1* (*VRN1*), CCT domain proteins (*VRN2*) and *FT* (*VRN3*) is involved in the vernalization response [121]. Before vernalization, *VRN3* induction in LDs is prevented by VRN2. Wheat VRN2 interacts with NF-Y complexes, similarly to the *A. thaliana* CCT domain protein CO [122]. Thus, it is possible that, also in grasses, chromatin loops are implicated in the regulation of VRN3 transcription, although experimental proof is lacking. As VRN2 is repressed by VRN1 that is, in turn, activated by VRN3, the gene network is bi-stable and expresses either *VRN2* or *VRN3–VRN1* [123]. The integration of chilling temperatures occurs at the level of *VRN1*, which gradually increases in expression during the cold, resulting in downregulation of *VRN2* in leaves. This allows *VRN3* to respond to LDs, leading to a ‘secondary’ strong upregulation of *VRN1*.

Barley and wheat variants not requiring vernalization have been selected to allow planting in spring. The dominant wheat TaVRN3-Hope allele is associated with a TE insertion in the promoter region that bypasses the repressive effect of VRN2 on *VRN3* [121]. In addition, a number of dominant *VRN1* alleles in spring wheat and barley cultivars are expressed before vernalization [123]. Small insertions/deletions (indels) and point mutations in the promoter as well as large indels in the first intron were associated with these alleles, and both regions are positive for H3K27me3 [124]. During the course of vernalization, H3K27me3 at *VRN1* slowly decreases, whereas H3K4me3 and H3 acetylation increase, concomitant with increased *VRN1* expression. The active chromatin state and increased *VRN1* levels are maintained after a return to warm temperatures. This could be attributable to an effect downstream of the induction of *VRN1* by VRN3 in LDs, the repression of *VRN2* in SDs or explained by an epigenetic memory due to the presence of TrxG complexes. From an evolutionary perspective, it is interesting to note that the vernalization memory system in cereals promotes an active, instead of a repressive, epigenetic state as in Brassicaceae. This exemplifies that evolution of vernalization memory systems cannot only involve different players of the genetic flowering time network but also engages ambient temperature-sensitive chromatin-modifying pathways in a versatile manner.

## Concluding remarks

Data from an increasing number of species are becoming available, and we find it helpful to consider evolutionary aspects to detect common principles and separate them from family-specific or species-specific complexity that can become quite confusing. A judicious choice of closely related annual and perennial species within the Brassicaceae has given insight into the connection between the different modules engaged in the regulation of flowering time. However, more models, in particular representing basic angiosperms and seed plants, could further advance our knowledge of the control of flowering. Vernalization pathways, as the latest addition to flowering time control, appear to recruit different key regulators and show considerable variation even within plant families. However, molecular memories implemented by PcG and TrxG complexes are a common denominator of the vernalization response in families as distant as Poaceae and Brassicaceae. Vernalization pathways evolved rapidly also in other families, where fewer molecular details are known. This rapid adaptation of plants to temperate climates could have been facilitated by an inherent sensitivity of chromatin and chromatin-modifying pathways to fluctuations in ambient temperature. The possibility to study and compare the response of chromatin to ambient temperature on a genome-wide and comparative level will answer whether, and to what extent, the chromatin is a holistic plant ‘thermometer’.

## Abbreviations

3C: chromatin conformation capture
5′-UTR: 5′-untranslated region
bHLH: basic helix-loop-helix
H2Bub: H2B mono-ubiquitylation
H3K4me3: histone H3 lysine 4 tri-methylation
H3K36me2/3: histone H3 lysine 36 di-/tri-methylation
indel: insertion/deletion
LD: long day
miRNA: micro-RNA
ncRNA: noncoding RNA
PcG: Polycomb group
PHD: pleckstrin-homology domain
PIC: pre-initiation complex
QTL: quantitative trait locus
RNAP II: RNA polymerase II
SD: short day
TrxG: Trithorax group
TSS: transcription start site
